# Self-reported changes in work situation – a cross-sectional study of patients 7 years after treatment for stress-related exhaustion

**DOI:** 10.1186/s12889-021-11242-5

**Published:** 2021-06-25

**Authors:** Anja Beno, Gunnel Hensing, Agneta Lindegård, Ingibjörg H. Jonsdottir

**Affiliations:** 1 Institute of Stress Medicine, Region Västra Götaland, Carl Skottsbergs gata 22B, SE-413 19 Gothenburg, Sweden; 2 School of Public Health and Community Medicine, Institute of Medicine, Sahlgrenska Academy, Gothenburg University, Gothenburg, Sweden

**Keywords:** Exhaustion disorder, Return to work, Working hours, Sick leave, Changes at work

## Abstract

**Background:**

Exhaustion disorder (ED) is a common cause of sick leave in Sweden, and patients often have long-lasting symptoms and reduced work capacity. The aim of this study was to explore whether patients with ED had made any changes in their work situation from the period of treatment and up to 7 years later.

**Methods:**

In this cross-sectional study, patients diagnosed with ED at a specialist outpatient clinic were followed up after 7 years (*n* = 217). They received questionnaires at baseline covering sex, age, marital status, level of education, and symptoms of burnout, depression, and anxiety measured with the Shirom-Melamed Burnout Questionnaire and the Hospital Anxiety and Depression Scale. After 7 years, they were sent a follow-up questionnaire asking about their work situation and work-related stressors both before they fell sick and at the 7-year follow-up. There were three questions on work situation (change of workplace, change of work tasks, and change of working hours), and 155 patients responded to all three.

**Results:**

After 7 years, the majority of the patients (63%; *n* = 98/155) reported that they had made some kind of change at work. Women were more likely than men to report decreased working hours (*p* = 0.001), and work-related stressors such as conflicts at work, reorganization, deficient leadership, and general discontent with the work situation were significantly more common at baseline in the group who had made changes at work. Patients who made no changes at work experienced more work-related stress due to quantitative demands in the 7-year follow-up.

**Conclusion:**

The majority of the patients with ED made some kind of change in their work situation, and gender differences were found for changes of work tasks and working hours. Work-related stressors might be decisive for making changes at work.

## Introduction

Mental disorders are the most common diagnoses relating to sick leave in Sweden. In 2019, 53% of women and 42% of men on sick leave had a psychiatric diagnosis. Within this group, the most common type of diagnosis was stress-related mental disorders: acute stress reaction, reaction to severe stress, and exhaustion disorder (ED) [1]. A recent review of burnout in the workplace indicates that increased sick leave due to stress at work is a general trend seen across Europe [2]. Sick leave among patients seeking care for symptoms of exhaustion due to prolonged exposure to psychosocial and work-related stress has been shown to be long-lasting [1, 3–5]. Diagnostic criteria for ED, which can be considered a clinical form of burnout [4], have been established in Sweden (Table 1).

**Table 1:** Diagnostic criteria for Exhaustion Disorder according to the National Board of Health and Welfare (2003)

A. Physical and mental symptoms of exhaustion with minimum two weeks duration. The symptoms have developed in response to one or more identifiable stressors which have been present for at least 6 months.	
B. Markedly reduced mental energy, which is manifested by reduced initiative, lack of endurance, or increase of time needed for recovery after mental efforts.	
C. At least four of the following symptoms have been present most of the day, nearly every day, during the same 2-week period:	
1. Persistent complaints of impaired memory.	
2. Markedly reduced capacity to tolerate demands or to work under time pressure.	
3. Emotional instability or irritability.	
4. Insomnia or hypersomnia.	
5. Persistent complaints of physical weakness or fatigue.	
6. Physical symptoms such as muscular pain, chest pain, palpitations, gastrointestinal problems, vertigo, or increased sensitivity to sounds.	
D. The symptoms cause clinically significant distress or impairment in social, occupational, or other important areas of functioning.	
E. The symptoms are not due to the direct physiological effects of a substance (e.g. drug abuse, medication) or a general medical condition (e.g. hypothyroidism, diabetes, infectious disease).	
F. If criteria for major depressive disorder, dysthymic disorder, or generalized anxiety disorder are met, exhaustion disorder is set as a comorbid condition.	

The main symptoms reported by patients with ED are exhaustion, cognitive dysfunction, sleep disturbance, reduced stress tolerance, and somatic symptoms [3, 6]. The burden of both mental and somatic symptoms is high, and similar for men and women [6]. Symptoms related to cognitive functions have been described in several studies, and these also seem to be long-lasting [7–9]. A recent long-term follow-up of patients who had previously sought care for ED found that as many as one third of patients were clinically defined to have exhaustion 7 years after seeking care. Furthermore, around 30–40% of the patients said that their memory and/or concentration was affected, and as many as 70% reported reduced stress tolerance even 7 years after first seeking care [10]. Persistent symptoms most likely affect work capacity, which raises the question of how well these individuals are functioning when returning to work, particularly those still fulfilling the criteria for ED.

The workplace is of central importance when discussing stress-related mental health problems. Thus, factors such as high demands and low control, high work load, low reward, and job insecurity, increase the risk of developing exhaustion [11]. We have previously shown that both work-related and non-work-related exposure are reported by patients with exhaustion disorder to contribute to their exhaustion. However, the far most commonly reported exposure reported by the patients is quantitative demands at work [12].

Furthermore, several studies focusing on return to work (RTW) for people with ED have found that involving the employer in the process seems to be the key for successful RTW, often with a particular focus of adjustment of work demands [13–15]. Thus, since poor psychosocial work environment, including high demands, has been shown to be a contributing factor to the exhaustion [12, 16], the need for changes regarding the work situation is relevant to discuss [17]. A recent study found that people with common mental disorders used different job crafting strategies related to the job task, relations at work, and cognitive function to fit their reduced work function [18]. Regarding job mobility, Liljegren at al. found that job mobility predicted better psychosocial health and less work-related burnout compared to remaining at the same workplace throughout the study period, but that burnout was not an primary reason for changing workplace [19]. In a 5-year follow up study of employment status among male production workers with sickness absence due to mental disorders, 18% of these workers left their employment but only 9% of workers without mental disorders did the same [20].

The development of exhaustion as well as rehabilitation and return to work among patients with stress-related exhaustion are interconnected with one of the most predominant work stress models within the field of psychosocial working conditions, namely the job demand-control (JDC) model [21] model as well as the extended model referred to as the job demand-control-support (JDCS) model [22]. Commonly the JDCS model has been used to measure psychosocial stress at workplaces but it can also serve as a reasonable model to be used to understand the driving forces for plausible changes at work, among individuals who have developed stress-related exhaustion.

If people have to make changes at work due to their illness, this affects the individual, the employer, and the labour market. It is therefore of great interest to explore how ED affects a patient’s work situation in the long term. However, to the best of our knowledge, there are no previous long-term studies of the extent to which people with stress-related exhaustion make changes at work, such as change of workplace and/or adjustments at work.

The primary aim of this study was thus to investigate whether patients with ED reported that they had made changes at work regarding workplace, work tasks, or working hours due to their exhaustion. The second aim was to investigate whether changes of work situation were related to sex, age, and symptoms of burnout, depression or anxiety. The third aim was to investigate whether changes at work were related to self-reported stress exposure.

## Methods

### Design

This exploratory cross-sectional study is part of a longitudinal cohort study and uses data from patients with ED collected 7 years after they were referred to a specialist clinic for exhaustion. The study was approved by the regional ethical review board in Gothenburg, Sweden, and conducted according to the 1964 Declaration of Helsinki.

### Participants

All participants had previously received treatment at a specialist tertiary outpatient clinic which exclusively treated patients with stress-related exhaustion, in the region of Västra Götaland, Sweden, between 2004 and 2012. The referral criteria to the clinic were stress-related exhaustion with no apparent somatic disorder or psychiatric diagnosis that could explain the exhaustion. The physicians at the clinic then ensured that the patient fulfilled the diagnostic criteria for ED before entering the treatment programme at the clinic. Patients with other diagnoses that could explain the symptoms were not treated at the clinic and thus not included in this study. The remitting physician had usually initiated some form of treatment. The diagnostic procedure and treatment for ED at the tertiary stress clinic is described elsewhere [3].

**Fig. 1:**
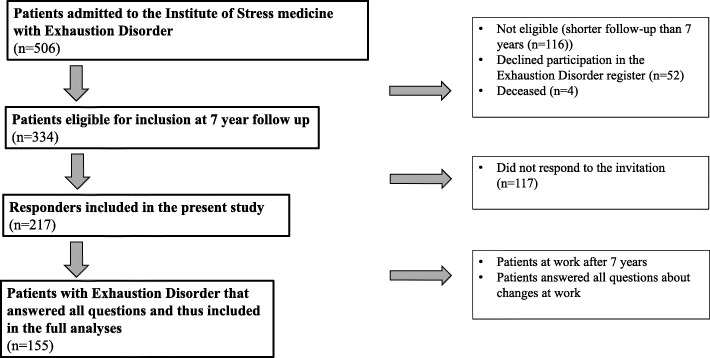
Flow chart of study inclusion. Of the 506 patients with exhaustion disorder admitted to the Institute of Stress Medicine, 334 were eligible for the 7-year follow-up, 116 were not eligible, 52 declined participation, and 4 had died. There were 217 responders (65%) and 117 non-responders to the 7-year follow-up questionnaires. Finally, 155 patients answered all questions about changes at work after 7 years

Following the treatment at the clinic, the patients were invited to participate in several follow-ups and asked to fill in a battery of questionnaires. Of the patients admitted to the specialist unit for treatment, 506 were followed up over time by register data. Data from patients who had reached their 7-year follow-up were included in this study. Of the 334 patients who were eligible for inclusion, 217 (65%) responded to the 7-year follow-up and were included in the present study. A subpopulation of 155 patients had full data on all questions regarding changes at work. A flow chart summarising participant inclusion is given in Fig. 1.

### Measurements

Baseline data (sex, age, marital status, level of education, and working hours) were collected during their first visit at the clinic. At their first visit to the clinic they also reported their current occupation and whether they were on sick leave.

#### Work situation

Current and former work situation was assessed by asking the patients what their work situation was at the time they sought care for their exhaustion, using five response options: 1. working full time/part time (%), 2. studying, 3. unemployed, 4. retired, and 5. parental leave full time/part time (%). The same five options were offered at the 7-year follow-up along with one extra: 6. no longer able to receive sickness benefits. Patients were also asked to state if they were currently on sick leave both at baseline and at the 7-year follow-up, using six response alternative: 1. not on sick leave, 2. sick leave 25%, 3 sick leave 50%, 4. sick leave 75%, 5. sick leave 100%, 6. disability pension.

Two questions were used to ask about changes at work: “Have you changed workplace because of your illness?” (yes/no) and “Have you changed your work tasks because of your illness?” (yes/no).

The number of individuals for each analysis varied due to missing values for some questions. Only individuals replying to all questions on work-related changes (i.e. changes in workplace, work tasks, and working hours) were included in the overall analysis of whether any changes had been made.

#### Symptoms of burnout, depression, and anxiety

The Shirom-Melamed Burnout Questionnaire (SMBQ) was used to measure symptoms of burnout at baseline [23]. It includes 22 items with response scales graded from 1 (almost never) to 7 (almost always). An analysis of its psychometric properties using the Rasch model resulted in a transformation from 22 items to 18, which was applied in this study [24]. Mean total Rasch scores for SMBQ were calculated (SMBQr), and a score above 4.40 was used as cut-off for burnout.

The Hospital Anxiety and Depression Scale (HAD) was used to assess symptoms of depression and anxiety at baseline [25]. It consists of two subscales of seven items each for depression and anxiety, giving a total of 14 items. Both subscales use the sum scores to classify “non-cases” (0–7), “possible cases” (7-10), and “cases” of depression and anxiety (> 10). In this study we dichotomized the answers into two groups: “non-cases” (0–10) and “cases” (11–20) for depression and anxiety.

#### Self-reported stressors

All participants were asked to fill in a questionnaire at follow-up asking about self-reported stress exposure that they themselves judged to be a contributor to their exhaustion, both currently and retrospectively. Thus, the patients were asked to state if the stressor had contributed to their exhaustion when they initially sought care, and if the stressor was currently contributing to a considerable strain for them today, 7 years after initially seeking care.

The stressors listed in the questionnaire were drawn from the results of a content analysis of medical records from the same group of patients [12]. In brief, during the clinical interview when the patients first visited the clinic, stressors were identified by the physician together with the patient as part of the diagnostic procedure and documented in the medical record. The content analysis of these records resulted in a total of 24 categories of stressors, of which 11 were related to work and 13 were non-work related. The work-related stressors are: Quantitative demands, Emotional demands, Conflicts at work, Management responsibility, Reorganization, Deficient leadership, Job insecurity, Irregular working hours, Traumatic events at work, Work environment and Discontent at work. The private stressors are: Death of family member, Caring for family member, Single parent, Relational conflicts, Separation, Change in family constitution, Worries for one’s health, Personal injury/illness, Financial worries, Residential stressor, Voluntary engagement, Legal matters and Loneliness.

An open alternative was also included in the questionnaire, giving the possibility of adding exposures not already listed.

### Data analysis

Age was dichotomized into younger than 40 years and 40 years or older. Marital status was dichotomized into married/living together and single/other, with the latter group including single, with a partner but living separately. Level of education was dichotomized into lower (elementary school/high school) and higher (university education). Patients on sick leave were dichotomized into those on part time sick leave (25, 50%, or 75%) and those on full time/permanent sick leave (100%). Working hours were divided into three groups: same working hours, reduced working hours, and increased working hours compared to the situation when they started treatment.

### Statistical analyses

Baseline descriptive statistics are given in terms of count and percentages. Pearson’s chi-square test was used to explore differences in sex, age, marital status, level of education, and comorbidity between those who answered the questionnaires and those who did not (drop-out analysis), as well as differences in sex, age, marital status, level of education, occupation, self-reported stressors, and symptoms of burnout, depression, and anxiety in the groups who changed workplace, work tasks, or working hours. Logistic regression analysis was used to evaluate whether sex, age, or symptoms of burnout (SMBQ), depression and anxiety (HAD) were associated with changes at work (i.e. change of workplace, work tasks, or working hours). The significance level was set at *p* < 0.05 and Holm-Bonferroni correction was used for multiple comparisons. All analyses were conducted using version 22 of IBM SPSS Statistics. Confidence intervals for differences in proportions for self-reported work-related stressors and changes at work were calculated by using Newcombe method 10 [26], using Epi package in R.

## Results

### Demographics at baseline

The characteristics of the patients at the beginning of treatment are summarized in Table 2.

**Table 2:** Baseline characteristics of patients with exhaustion disorder included in the study (*n* = 217) compared with eligible non-responders (*n* = 117)

	Total *n* = 334	Responders n (%)	Non-responders n (%)	***p***-value
		217 (65%)	117 (35%)	
**Sex**				0.08
Women	237 (71%)	161 (74%)	76 (65%)	
Men	97 (29%)	56 (26%)	41 (35%)	
**Age**				0.08
< 40 years	130 (39%)	77 (36%)	53 (45%)	
≥ 40 years	204 (61%)	140 (64%)	64 (55%)	
**Marital status**				0.64
Married/living together	242 (73%)	159 (74%)	83 (72%)	
Single/other	89 (27%)	56 (26%)	33 (28%)	
**Education**				0.57
Lower	105 (32%)	66 (31%)	39 (33%)	
Higher	227 (68%)	150 (69%)	77 (66%)	
**Burnout**				
**SMBQr**				0.11
< 4.39	28 (9%)	22 (11%)	6 (5%)	
≥ 4.40	289 (91%)	184 (89%)	105 (95%)	
**Comorbidity**				
**HAD depression**				0.79
0–10	213 (65%)	137 (64%)	76 (65%)	
11–20	117 (35%)	77 (36%)	40 (35%)	
**HAD anxiety**				0.17
0–10	112 (34%)	78 (37%)	34 (29%)	
11–20	218 (66%)	135 (63%)	83 (71%)	

Analyses for each group that did/did not answer the questionnaires were performed for sex, age, marital status, level of education, and symptoms. Pearson’s chi-square was used for all tests, and *p* < 0.05 was considered to be significant

*SMBQ* Shirom-Melamed Burnout Questionnaire Rasch score, *HAD* Hospital Anxiety and Depression Scale

Of those included in this study, 74% (*n* = 161/217) were women, 26% (*n* = 56/217) were men, and the majority (74%; *n* = 159/217) were married/living together. The mean age when initially seeking care was 43.76 years (SD: 9.22; range: 22–64) for the whole group.

### Prevalence of changes at work

At the 7-year follow-up, 71% of the patients (*n* = 155/217) answered all three questions regarding their work situation; that is, whether they had changed their workplace, work tasks, and/or working hours. Most of them (63%; *n* = 98/155) reported that they had made some kind of change at work with regard to at least one of these three aspects, and so only 37% (*n* = 57/155) reported being in the same work situation regarding workplace, work tasks, and working hours. Of those who reported that they had made some kind of change at work, 43% (*n* = 67/155) had made changes in more than one of the three aspects. At baseline, 61% (132/217) were on full time sick-leave, 21% (45/217) were on part-time sick-leave and 18% (40/217) were not on sick-leave. At the 7-year follow-up, 3% (7/217) were on full time sick-leave, 4% (8/217) had received sickness pension, 6% (12/217) were on part time sick-leave and 87% (188/217) were not on sick-leave. Sick-leave data was missing for 2 individuals at 7 years follow-up.

### Change of workplace and/or work tasks

Self-reported changes of workplace and work tasks are shown in Table 3. Overall, 47% of the patients (*n* = 91/194) reported that they had changed workplace. No differences were found between the groups regarding sex, age, marital status, level of education, or symptoms of burnout, depression, or anxiety between. Similarly, 42% (*n* = 80/192) reported that they had changed their work tasks because of their illness. Men were more likely than women to have changed their work tasks (*p* = 0.03), but otherwise the groups did not differ (Table 3). After the Holm-Bonferroni correction the difference between men and women was no longer significant.

**Table 3:** Changes at work in a 7-year follow-up of patients with exhaustion disorder

	Change of workplace n (%)		p	Change of work tasks n (%)		p	Reduced working hours n (%)		p
	yes	no		yes	no		yes	No	
**Sex**			0.32			0.03^b^			**0.001**
women	64 (45%)	79 (55%)		52 (37%)	89 (63%)		33 (31%)	75 (69%)	
men	27 (53%)	24 (47%)		28 (55%)	23 (45%)		2 (4%)	45 (96%)	
**Age**			0.73			0.85			0.68
< 40 years	10 (44%)	13 (56%)		10 (44%)	13 (56%)		4 (19%)	17 (81%)	
≥ 40 years	81 (47%)	90 (53%)		70 (41%)	99 (59%)		31 (23%)	103 (77%)	
**Marital status**^**a**^			0.62			0.06			0.42
married/living together	66 (46%)	78 (54%)		54 (38%)	89 (62%)		29 (24%)	91 (76%)	
single/other	24 (50%)	24 (50%)		25 (53%)	22 (47%)		6 (18%)	28 (82%)	
**Education**^**a**^			0.83			0.56			0.95
lower	27 (46%)	32 (54%)		26 (45%)	32 (55%)		10 (22%)	35 (78%)	
higher	64 (47%)	71 (53%)		54 (40%)	80 (60%)		25 (23%)	85 (77%)	
**Comorbidity**									
**SMBQr**			0.32			0.14			0.36
< 4.39	72 (51%)	68 (49%)		58 (42%)	81 (58%)		25 (21%)	96 (79%)	
≥ 4.40	17 (43%)	23 (57%)		22 (55%)	18 (45%)		7 (29%)	17 (71%)	

Analyses for each group (change of workplace and change of work tasks) were performed to explore whether there were any significant differences between the groups in terms of sex, age, marital status, level of education, and symptoms of exhaustion disorder. Pearson’s chi-square was used for all tests, and *p* < 0.05 was considered to be significant

*SMBQ* Shirom-Melamed Burnout Questionnaire Rasch score

^a^Baseline data

^b^After the Holm-Bonferroni correction the difference between men and women was no longer significant

### Changes in working hours

**Fig. 2:**
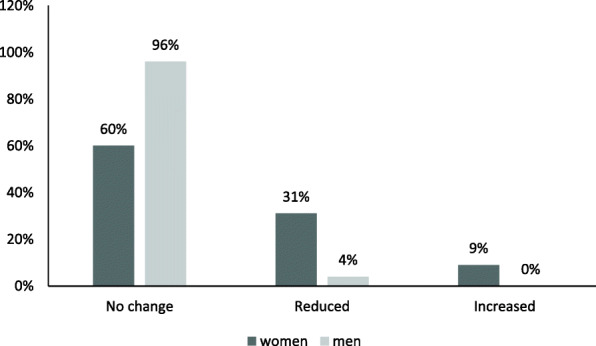
Changes in working hours and differences between women and men at the 7-year follow-up among exhaustion disorder patients

Most of the patients (71%; *n* = 110/155) had the same working hours as before they first sought care, whereas around a quarter (23%; *n* = 35/155) had reduced their working hours and a few (7%; *n* = 10/155) had increased them. Women were more likely than men to have reduced their working hours (*p* = 0.001; Fig. 2). Holm-Bonferroni correction did not change the significance.

Age, marital status, level of education, and symptoms of burnout were not related to changes in working hours (Table 3)*.*

### Association between changes at work and sex, age, and symptoms of burnout

Bivariate logistic regression analysis was used to examine associations between sex, age, symptoms of burnout at baseline (covariates), and change of workplace, change of work tasks, and reduced working hours at the 7-year follow up. The odds for reduced working hours were higher for women than for men (OR: 0.06, 95% CI: 0.008–0.46), while the odds for change of work task were higher for men than for women (OR: 2.29, 95% CI: 1.15–4.56). There was no association between the covariates and change of workplace (Table 4).

**Table 4:** Logistic regression analysis examining the association between sex, age, symptoms of burnout, and changes at work

	Change of workplace	Change of work tasks	Reduced working hours
	*OR (95% CI)*	*OR (95% CI)*	*OR (95% CI)*
**Age**	0.95 (0.51–1.76)	1.44 (0.76–2.72)	0.79 (0.35–1.81)
**Sex**	1.46 (0.74–2.89)	2.29^*^ **(1.15–4.56)**	0.06^**^ **(0.008–0.46)**
**SMBQ**	2.22 (0.79–6.25)	1.06 (0.40–2.91)	0.95 (0.27–3.35)
**HADdep**	1.19 (0.61–2.35)	1.47 (0.73–2.95)	1.40 (0.56–3.49)
**HADanx**	1.00 (0.51–1.93)	0.79 (0.40–1.55)	1.07 (0.44–2.61)

Logistic regression analysis examining the association between sex, age, symptoms of burnout (SMBQ),

and changes at work (i.e. change of workplace, change of work tasks, and reduced working hours)

* *p* = 0.02, **p = 0.006.

*OR* odds ratio, *CI* confidence interval, *SMBQ* Shirom-Melamed Burnout Questionnaire, *HAD* Hospital anxiety and depression scale

### Stressors and changes at work

In this part of the analysis, we examined associations between self-reported stressors and changes at work in two ways: first, whether stressors retrospectively reported to be a contributing cause of illness at baseline were associated with changes at work, and second, whether stressors still reported at the 7-year follow-up were associated with changes at work. Data from 130 patients were available for these analyses. In comparison to those who had made no changes, a significantly higher percent of patients who had made some kind of change at work reported baseline stress exposure related to conflicts at work, reorganization, deficient leadership, and discontent regarding the overall work situation. The groups reported a similar pattern of current stressors at the 7-year follow-up, except that those who had made no changes at work reported significantly more stress due to quantitative demands at work (Table 5). Among the private stressors, only relational conflicts were reported significantly higher among patients who made changes at work at baseline 19.8%, CI (− 37.6:-0.5), after 7 years patients who made no changes at work reported significantly more relational conflicts − 14.9%, CI (− 31.5:-0.1).

**Table 5:** Self-reported work-related stressors at baseline (TI) and at the 7-year follow-up (T2) (*N* = 130)

Work stressors	Time	Changes at work % (n)	No changes at work % (n)	Difference in percentages (95% CI)
Quantitative demands	T1	87 (61)	84 (32)	2.9 (−9.9; 18.7)
	T2	34 (24)	63 (24)	−28.9 (−45.7; −9.2) ^a^
Emotional demands	T1	64 (45)	69 (26)	−4.1 (− 21.3; 14.7)
	T2	24 (17)	42 (16)	−17.8 (−35.7; 0.3)
Conflicts at work	T1	53 (37)	29 (11)	23.4 (4.3; 40.3) ^a^
	T2	16 (11)	11 (4)	5.2 (−10; 17.3)
Management responsibility	T1	43 (30)	32 (12)	11.3 (−8; 28.4)
	T2	10 (7)	16 (6)	−5.8 (−21.3; 6.7)
Reorganization	T1	53 (37)	27 (10)	26.5 (7.1; 42.5) ^a^
	T2	23 (16)	17 (15)	−16.6 (−34.5; 1.1)
Deficient leadership	T1	70 (49)	25 (17)	25.3 (5.9; 42.7) ^a^
	T2	19 (13)	37 (14)	−18.3 (−35.8; 1.1)
Job insecurity	T1	17 (12)	5 (2)	−11.9 (−2.1; 23)
	T2	11 (8)	11 (4)	0.9 (−13.8; 12.4)
Irregular working hours	T1	36 (25)	21 (8)	14.7 (−3.7; 30)
	T2	10 (7)	13 (5)	−3.2 (−18.2; 8.7)
Traumatic events at work	T1	24 (17)	11 (5)	11 (−5.4; 24.6)
	T2	4 (3)	4 (3)	−3.6 (−16.8; 5.6)
Work environment	T1	27 (19)	29 (11)	−1.8 (−20; 14.7)
	T2	10 (7)	32 (12)	−21.6 (−38.2; −6)
Discontent at work	T1	39 (27)	5 (2)	33.3 (17.3; 45.6) ^a^
	T2	3 (2)	5 (2)	−2.4 (−14.6; 5.5)

Self-reported work-related stressors at baseline (TI) and at the 7-year follow up (T2) were compared between the group who made changes at work and the group who made no such changes, in order to explore significant differences between the groups at baseline and at the 7-year follow-up

^a^Significant difference

## Discussion

The main finding of this study was that the majority, around 60%, of patients with previous exhaustion disorder reported that they had made some kind of change in their work situation as a consequence of the exhaustion, including either changing their workplace, changing their work tasks, and/or changing their working hours. Almost half of the patients had changed their workplace, more than 40% had changed their work tasks, and almost 25% had reduced their working hours. One important finding was that sex was the sole predictor for changes at work, with women being more likely to reduce their working hours and a trend for men being more likely to change work tasks.

Thus, around half of the population included in this study reported that they had changed workplace due to their illness. Putting these results into perspective, between 1994 and 2008 the general Swedish labour market mobility in the form of change of workplace within 1 year varied between 13 and 19%. A general population study performed in the same region, as our study found that over a 5-year period 25.5% of participants changed their workplace [27]. Individuals with and without sick leave experience seemed to change their workplace to a similar degree [28]. The general mobility on the labour market in Sweden is relatively low compared to other countries, due to historical commitment to full employment and the so-called Swedish ‘work line’ which aims at protecting employment as such rather than particular jobs [29]. Mobility close to 50% found in this study could therefore be considered relatively high, particularly since sick leave in general is not related to change of workplace [28].

A 5-year follow-up study of production workers with sickness absence due to mental disorders reported that 18% left their employment during this period [20]. Similar job mobility was found in a study on patients with mild traumatic brain injury, showing that 17.3% of the patients had exited their employment and 15.5% needed to make changes at work to enable to continue their employment 4 years post-injury [30]. Patients with common mental disorders and mild traumatic brain injury have similar symptoms to patients with ED, in terms of cognitive deficits and mental fatigue. Thus, we can cautiously speculate that the burden of symptoms does not seem to be the main driving force behind changes at work, even though it is most probably part of the reason. Furthermore, patients with common mental disorders and patients with ED are likely to relate differently to their work environment compared to, for example, patients with stroke; this includes the process of RTW, since work-related factors are often strongly related to these mental conditions [11, 31].

Thus, given the above, it is likely that patients with ED were forced to make changes at work partly due to remaining symptoms but also due to poor psychosocial work environment [32, 33]. Indeed, the group that did make changes in their work situation reported a somewhat poorer work situation than those who made no such changes, regarding conflicts at work, reorganization, deficient leadership, and general discontent with their work situation. These types of work-related stressors are known to contribute to burnout, and it could be speculated that they might also contribute to a decision to make changes at work [11]. It can thus be hypothesized that one contributing factor to making changes in the work situation was the psychosocial work environment, including high job demands, which thus plausible have not changed during the time that the patients were off work. The group who made changes reported a higher degree of conflict at the workplace; and regardless of the reason for this conflict, it might have been difficult for these individuals to return to the same workplace. Other plausible reasons for changes in the work situation could include feelings of shame about becoming ill and/or private related situations.

Another important finding was that patients who had made some kind of change at work reported significantly more stressors at baseline related to conflicts at work, reorganization, deficient leadership, and discontent compared to the group who had not made any such changes. To the best of our knowledge, no previous study has explored changes in work situation among individuals who have been on sick leave due to stress-related exhaustion.

Thus, those who have made changes describe a psychosocial work situation at baseline that is clearly linked to higher psychological strain including situation such as conflicts and reorganisations as well as factors such as deficient leadership suggesting lack of support. According to the JDCR model, high demands and lack of resources are factors that have contributed to the exhaustion resulting in sickness absence. The job resource part of the model has on the other hand been shown to be related to work engagement and intention to stay at the workplace [34]. We can thus speculate that the individuals that choose to make changes at work still experiences the work situation to be unsatisfying, particular with regard to resources which affects their work engagement and motivation to stay at the same workplace [34]. If changes to avoid stress at work are made, this would plausibly lead to gain resources at work and reduce stress exposure and thus also the risk for stress-related exhaustion.

This is in line with the results showing that the group that had made no changes at work reported significantly more work-related stress after 7 years due to quantitative demands and poor working environment, indicating that changes in the psychosocial work environment are needed in these workplaces. Private related stress was not clearly associated with changes at work except from relational conflicts, indicating that changes at work is mainly associated with work-related stressors. Surprisingly, no difference was seen with regard to remaining symptoms between patients who still experienced work-related stress and those who did not. It could be expected that productivity was affected in patients who were still experiencing a poor psychosocial work environment, but this was not measured in the current study.

Changes in work tasks are plausibly related to difficulties stemming from remaining symptoms. It has previously been shown that as many as one third of patients with ED still fulfil the clinical criteria after 7 years, and many still report problems with cognitive function, fatigue, and reduced stress tolerance [10]. This could also be the primary reason for reducing working hours, though other reasons such as making different priorities in life and gaining a balance between work and leisure are also plausible.

The social insurance system in Sweden might also be of importance to discuss as a possible explanation for changes at work, since many patients with ED are on sick leave at some point. In the present system, a workplace transfer to another job with the same employer but with lesser demands must take place after 3 months. Furthermore, according to the regulations, a discussion regarding work ability and plausible return to the same workplace should be conducted after 6 months [35]. Several aspects related to the regulations on sick-leave benefits, such as time pressure, uncertainty regarding work ability, position at work, and financial situation, could also plausibly contribute to pressure on both the employer and the individual to make changes that otherwise would not have been made.

### Gender predicts changes at work

In this study, we found that women were more likely to reduce their working hours while men tend to be more likely to change their work tasks when analysed in the regression model. This difference in changes at work raises several questions. Previous studies found that there was no difference in burden of symptoms between women and men with ED [6], and thus it is not likely that women and men make different changes at work due to their burden of symptoms. A more plausible explanation could be gender segregation on the labour market; women and men generally work in different sectors, and gender segregation is also seen even within the same sector [36]. In general, women more frequently work part time, use most of the insurance system for parental leave, and spend more time on domestic work [36]. Thus, one explanation for why women in this study tended to reduce their working hours to a larger extent than men could be that they performed more domestic work and had less time for recovery in their private life. In a societal perspective men historically have been considered to be the breadwinners and in many countries women still are more likely to work part-time than men [37].

The gender difference regarding working hours is somewhat problematic, since women already have a less favourable situation when it comes to income, with lower lifetime income and pension than men [38, 39]. Women are more commonly employed in the educational and healthcare sectors, where it might be more difficult to change or adjust work tasks. Men, on the other hand, have professions such as technician and craftsman, where it might be easier to change or adjust work tasks [38]. A previous study found gender differences in rehabilitation, with men being more likely than women to demand actions and to strive for full-time waged work [40]. It might also be easier to make adjustments at work in sectors which mainly employ men than in sectors which mainly employ women.

### Limitations and strengths

There are several limitations in this study that need to be considered. The patients included were admitted to a specialist tertiary clinic, and might thus have had a higher burden of symptoms compared to what might be expected within primary care [41]. Most of them had higher education, which might have affected their ability to make changes at work. Relatively few men were included in the study, and so conclusions regarding differences between men and women need to be confirmed in larger studies. It was not possible to make direct comparisons with a working population in this study, and so we cannot draw conclusions regarding whether changes in work situation are more common among patients with ED compared to the general working population.

All data on changes at work were retrospective self-reports, using register data on employment status would have given more accurate information. The questions about changes of workplace and work tasks specifically asked if changes were made because of their illness or if the patients were forced to make changes due to other reasons. Working hours were reported at baseline and at the 7-year follow-up, but we did not ask specifically if participants had changed their working hours due to their illness; this is an important limitation of this study, and so these results must be interpreted with caution. Another limitation of this study is that we did not ask for details of when they made the changes at work during the 7-year period. Changes at work could have been done at different points during the past 7 years and the symptoms of ED, depression and anxiety might have fluctuated over the past 7 years and possible affected changes at work.

Another methodological consideration that should be mentioned is that work-related stressors were measured as self-reports at 7-year follow up and thus plausible recall bias must be considered as a limitation.

### Clinical implications and further perspectives

A majority of patients with exhaustion disorder made considerable changes regarding their work situation, and it is plausible to believe that their psychosocial work environment has been a major trigger for these changes. The role of the workplace in the RTW process is of utmost importance, and good collaboration between the healthcare provider and the workplace needs to be established. However, most sick-leave notes for stress-related mental health problems are dispensed by general practitioners within the primary health care system. Thus, it is important to increase the knowledge of the importance of involving the workplace in the rehabilitation process. Adjustments at the workplace are in many cases both necessary and favourable and might avoid personal turnover and recurrent sick leave. Nevertheless, forced changes due to deficient support in the RTW process or due to regulations in the insurance system can also drive competent people to change to workplaces where they are unable to use their education and/or knowledge, resulting in a great loss of competence in the workforce. Further research is needed to understand why people with ED make changes at work.

Our study also illustrates gender differences in the rehabilitation process for patients with ED; awareness of this is particularly important in order to avoid further marginalization of women on the labour market.

## Conclusion

Patients with ED seem to make considerable changes in their work situation. Aspects of work-related stress such as conflicts at work, reorganization, deficient leadership, and general discontent at work were more common among those who made changes and might thus be decisive for making changes. An important gender difference was noted that needs to be considered in the return to work process of patients with exhaustion disorder, ensuring the same support for both women and men.

## Data Availability

The datasets generated and analysed during the current study are not publicly available due to that this data is a part of a larger dataset which is still being analysed and thus not yet published but data is available from the corresponding author on reasonable request”.

## Abbreviations

ED: Exhaustion Disorder
RTW: Return to work
SMBQ: Shirom-Melamed Burnout Questionnaire
HAD: Hospital Anxiety and Depression Scale
